# Fast solubilization of recalcitrant cellulosic biomass by the basidiomycete fungus *Laetisaria arvalis* involves successive secretion of oxidative and hydrolytic enzymes

**DOI:** 10.1186/s13068-014-0143-5

**Published:** 2014-10-08

**Authors:** David Navarro, Marie-Noëlle Rosso, Mireille Haon, Caroline Olivé, Estelle Bonnin, Laurence Lesage-Meessen, Didier Chevret, Pedro M Coutinho, Bernard Henrissat, Jean-Guy Berrin

**Affiliations:** INRA, UMR1163 Biotechnologie des Champignons Filamenteux, 13288 Marseille, France; Aix-Marseille Université, Polytech Marseille, UMR1163 Biotechnologie des Champignons Filamenteux, 13288 Marseille, France; CIRM-CF, UMR1163 Biotechnologie des Champignons Filamenteux, 13288 Marseille, France; INRA, Unité de Recherche Biopolymères, Interactions, Assemblages, 44316 Nantes, France; INRA, UMR1319 Micalis, Plateforme d’Analyse Protéomique de Paris Sud-Ouest, 78352 Jouy-en-Josas, France; CNRS, UMR7257 Architecture et Fonction des Macromolécules Biologiques, 13288 Marseille, France; Aix-Marseille Université, UMR7257 Architecture et Fonction des Macromolécules Biologiques, 13288 Marseille, France; Department of Biological Sciences, King Abdulaziz University, Abdullah Sulayman, Jeddah, 22254 Saudi Arabia

**Keywords:** Cellulose, Filamentous fungi, Carbohydrate-active enzymes, Bioenergy, Biorefinery, Lytic polysaccharide monooxygenase (LPMO)

## Abstract

**Background:**

Enzymatic breakdown of lignocellulosic biomass is a known bottleneck for the production of high-value molecules and biofuels from renewable sources. Filamentous fungi are the predominant natural source of enzymes acting on lignocellulose. We describe the extraordinary cellulose-deconstructing capacity of the basidiomycete *Laetisaria arvalis*, a soil-inhabiting fungus.

**Results:**

The *L. arvalis* strain displayed the capacity to grow on wheat straw as the sole carbon source and to fully digest cellulose filter paper. The cellulolytic activity exhibited in the secretomes of *L. arvalis* was up to 7.5 times higher than that of a reference *Trichoderma reesei* industrial strain, resulting in a significant improvement of the glucose release from steam-exploded wheat straw. Global transcriptome and secretome analyses revealed that *L. arvalis* produces a unique repertoire of carbohydrate-active enzymes in the fungal taxa, including a complete set of enzymes acting on cellulose. Temporal analyses of secretomes indicated that the unusual degradation efficiency of *L. arvalis* relies on its early response to the carbon source, and on the finely tuned sequential secretion of several lytic polysaccharide monooxygenases and hydrolytic enzymes targeting cellulose.

**Conclusions:**

The present study illustrates the adaptation of a litter-rot fungus to the rapid breakdown of recalcitrant plant biomass. The cellulolytic capabilities of this basidiomycete fungus result from the rapid, selective and successive secretion of oxidative and hydrolytic enzymes. These enzymes expressed at critical times during biomass degradation may inspire the design of improved enzyme cocktails for the conversion of plant cell wall resources into fermentable sugars.

**Electronic supplementary material:**

The online version of this article (doi:10.1186/s13068-014-0143-5) contains supplementary material, which is available to authorized users.

## Introduction

Lignocellulosic biomass is recognized as a sustainable source of mixed sugars for fermentation to second generation biofuels and biomaterials [1]. Its resistance to enzymatic deconstruction, however, is a major bottleneck for the development of cost-effective biorefineries [2]. Currently, high enzyme loadings of cellulases are needed because of their low specific performance (or activity) compared to that of other polysaccharide-degrading enzymes. In order to achieve sustainable biomass deconstruction into fermentable sugars it is necessary to overcome the chemical and structural complexity of biomass through the development of more efficient enzyme preparations.

In industry, the fungus *Trichoderma reesei* has been established as the major workhorse for the production of cellulases for second generation biorefineries [3]. In spite of remarkable cellulolytic ability, the genome of *T. reesei* has revealed a reduced set of lignocellulose-acting enzymes compared to other saprotrophic fungi [4]. *T. reesei* is also particularly poor in lytic polysaccharide monooxygenases (LPMOs), the addition of which was shown to increase the saccharification of biomass [5] through the oxidative cleavage of cellulose [6].

In nature, lignocellulose is primarily degraded by wood and litter decomposers [7,8]. White-rot fungi have the enzymatic machinery required to completely breakdown all plant cell wall components (cellulose, hemicelluloses and lignin). In contrast, brown-rot fungi possess the capacity to depolymerize cellulose through the action of highly reactive hydroxyl radicals, leaving lignin behind as a slightly modified residue [9,10]. Basidiomycete decomposers of litter (litter-rots) have received less attention for their ability to breakdown lignocellulose, although they are known to act on partially degraded plant materials that are highly recalcitrant to enzymatic degradation [11].

Here, we provide insights on the remarkable biomass-degrading capabilities of the basidiomycete fungus *Laetisaria arvalis* (*Aphyllophorales, Corticiaceae*). By combining biochemical assays, transcriptomics and proteomics, we analyzed the temporal and substrate-dependent modulation of the enzymes recruited by this promising fungus for efficient and extended plant biomass deconstruction.

## Results

### Rapid growth on biomass and cellulose

**Figure 1:**
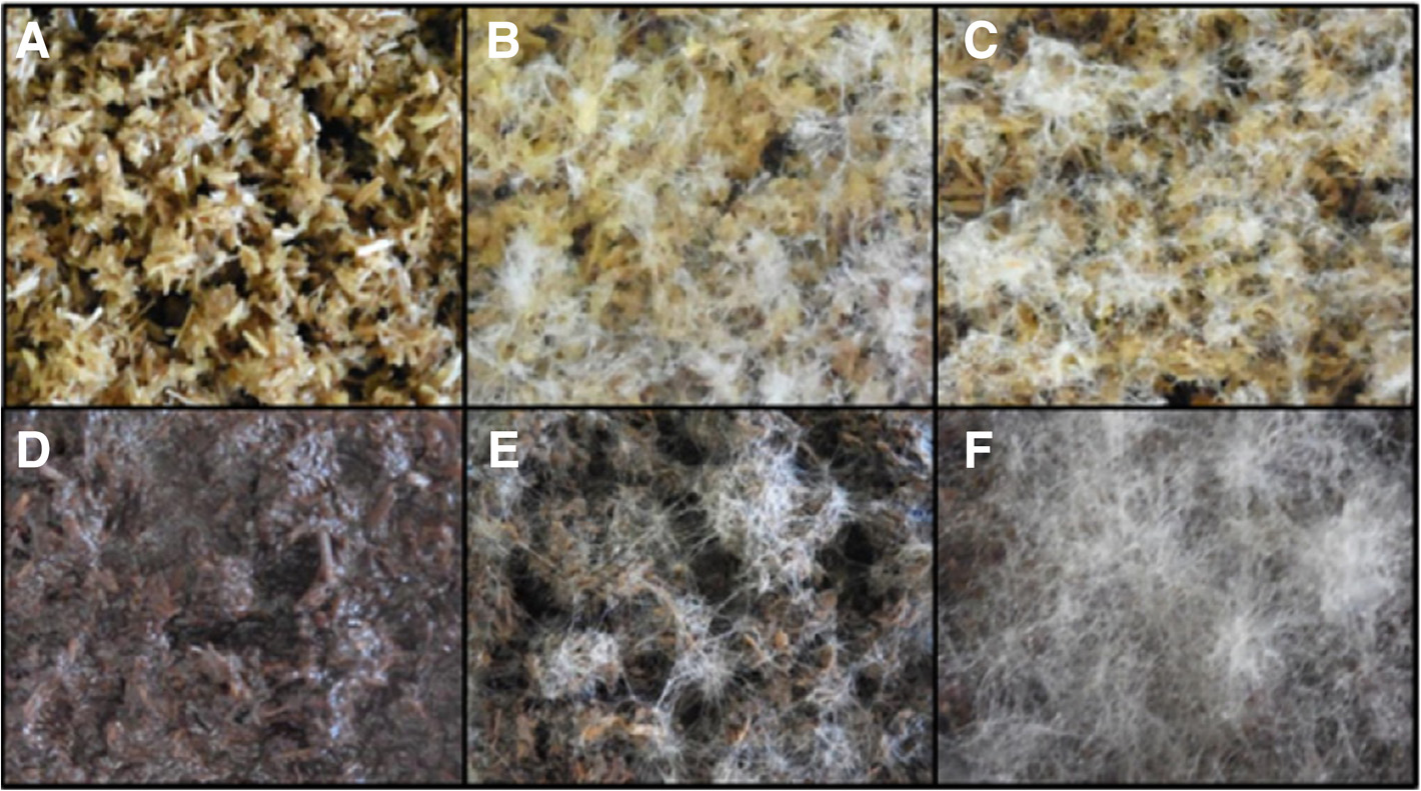
**Evaluation of the growth capabilities of** ***Laetisaria arvalis*** **on biomass** ***.*** Solid-state cultures on wheat straw **(A,B,C)** and wheat straw residue (WS-R) resulting from steam explosion under acidic conditions and subsequent saccharification with a *Trichoderma reesei* enzyme cocktail **(D,E,F)** after two days **(B,E)** and four days **(C,F)**. A and D represent controls without inoculum. These results are representative of several independent experiments.

**Figure 2:**
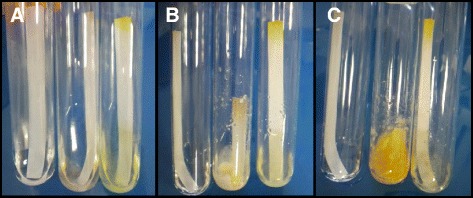
**Evaluation of the cellulose-degrading capabilities of** ***Laetisaria arvalis.*** Growth was performed in minimal medium containing filter paper strips as the sole carbon source. Pictures were taken after one day **(A)**, five days **(B)** and 10 days **(C)**. Left tube: control without inoculum; middle tube: *L. arvalis*; right tube: *Trichoderma reesei* QM6a. These results are representative of several independent experiments.

The *L. arvalis* strain CIRM-BRFM514 was identified when screening fungal strains for their capacity to efficiently degrade lignocellulosic biomass [12,13]. This strain displayed a great capacity to grow on wheat straw (WS) and on the leftover WS residue (WS-R) resulting from steam explosion under acidic conditions and subsequent saccharification (enzymatic conversion into sugars) with a *T. reesei* enzyme cocktail. The difference between WS and WS-R in terms of sugar composition is described in Additional file 1: Table S1. Indeed, the mycelium of *L. arvalis* fully colonized Petri dishes after only four days growth on WS and WS-R as the sole carbon source (Figure 1). *L. arvalis* was also able to grow and fully digest filter paper strips soaked in minimal medium within 10 days (Figure 2). In the same conditions, the wild-type *T. reesei* strain QM6a grew without visible breakdown of cellulose (Figure 2).

### *Laetisaria arvalis* has a complete set of lignocellulose-active enzymes

Given that no genomic data was available for fungi from the genus *Laetisaria*, and that the *L. arvalis* strain displays heterokaryotic multinucleate cells (Additional file 1: Figure S1) that would hamper correct genome assembly, we sequenced the global transcriptome of *L. arvalis* in order to explore its lignocellulolytic enzymatic capabilities. The 454 sequencing runs resulted in 367,307 reads (Additional file 1: Figure S2A). A total of 240,696 reads were assembled into 47,516 contigs (including 31,837 short read singletons). The transcriptome data are available at the European Bioinformatics Institute Sequence Reads Archive under accession number [EMBL:SRP041278]. The 15,679 assembled contigs (available in Additional file 2: Data S1) displayed an average length of 1,185 nucleotides (Additional file 1: Figure S2B). Transcriptome of gene ontology (GO) annotation using BlastX was performed using Blast2GO tools (June 2013) and it resulted in the identification of significant hits for 9,680 contigs, for which sequence similarity values ranged from 33 to 100%, with an average identity to reference sequences around 70% (Additional file 1: Figure S3A). For each query sequence, the top hit was used to give insights into relatedness with fully sequenced fungal species. The best hits were found in *Punctularia strigosozonata* (2,536 hits), followed by *Serpula lacrymans* (909 hits) (Additional file 1: Figure S3B).

**Figure 3:**
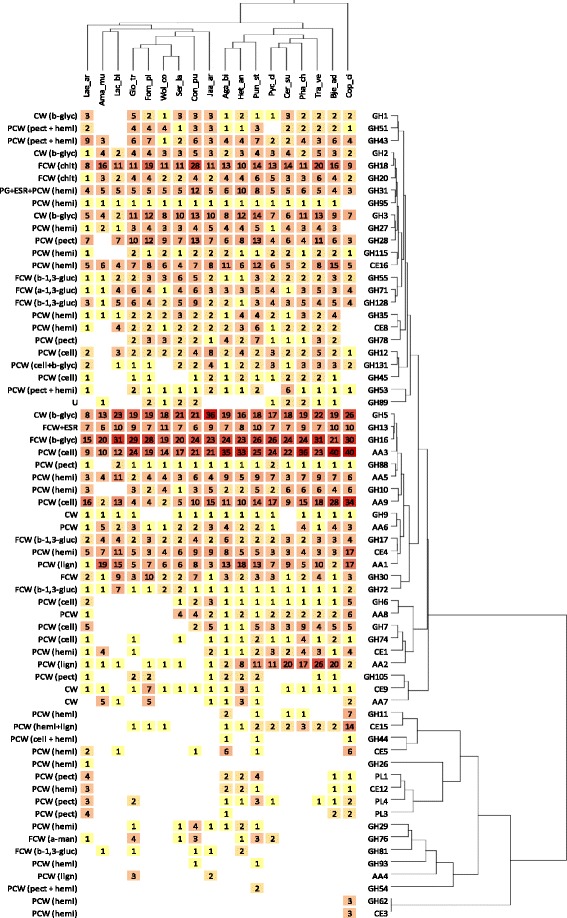
**Comparison of** ***Laetisaria arvalis*** **CAZymes to other fungi.** The CAZyme sets identified in the genomes of selected fungi and the transcriptome of *L. arvalis* CIRM-BRFM514 were compared using double hierarchical clustering. Top tree: fungal genomes analyzed : Lae_ar, *Laetisaria arvalis*; Lac_bi, *Laccaria bicolor*; Glo_tr, *Gloeophyllum trabeum*; Fom_pi, *Fomitopsis pinicola*; Wol_co, *Wolfiporia cocos*; Ser_la, *Serpula lacrymans*; Con_pu, *Coniophora puteana*; Aga_bi, *Agaricus bisporus var. burnettii*; Het_an, *Heterobasidion annosum;* Pun_st, *Punctularia strigosozonata*; Pyc_ci, *Pycnoporus cinnabarinus*; Cer_su, *Ceriporiopsis subvermispora*; Pha_ch, *Phanerochaete chrysosporium*; Tra_ve, *Trametes versicolor*; Bje_ad, *Bjerkandera adusta*; Cop_ci, *Coprinopsis cinerea*. Right tree: enzyme families represented by their class (GH, GT, PL, CE, CBM and AA) and family number according to the carbohydrate-active enzyme database. The double hierarchical clustering was performed using the Gingko Multivariate Analysis System [15]. Known substrates of CAZy families (most common forms in brackets) are indicated to the left: CW, cell wall; ESR, energy storage and recovery; FCW, fungal cell wall; PCW, plant cell wall; PG, protein glycosylation; U, undetermined; a-1,3-gluc, α-1,3-glucans; a-man, α-mannans; b-1,3-gluc, β-1,3-glucan; cell, cellulose; chit, chitin/chitosan; hemi, hemicelluloses; lign, lignin; pect, pectin.

The 15,679 assembled contigs were assigned to carbohydrate-active enzyme (CAZyme) families listed in the CAZy database [14]. Overall, the assembled transcriptome yielded a total of 266 CAZymes. Because the number of glycosyltransferases (GTs) is comparable among fungi, the completeness of the *L. arvalis* global transcriptome was evaluated by comparing the number of GTs to that found in sequenced basidiomycete genomes. *L. arvalis* displayed a number of GTs similar to that encoded by the genomes of other fungi (Additional file 1: Figure S4), suggesting that the global transcriptome successfully captured most CAZyme-encoded genes. The composition and richness of each CAZyme family revealed a complete set of CAZymes covering most of the families, with a total of 149 glycoside hydrolases (GHs), 15 polysaccharide lyases (PLs), 22 carbohydrate esterases (CEs), 32 auxiliary activity enzymes (AAs) and 48 carbohydrate-binding modules (CBMs). The uniqueness of *L. arvalis* was revealed when comparing its CAZyme repertoire with taxonomically related fungi (Figure 3). *L. arvalis* displayed a large set of enzymes from families GH7 and AA9, with five and 16 members, respectively. This was completed by a significant set of other predicted cellulose-degrading enzymes from (sub)families GH5_5, GH6, GH45, GH74 and GH131. Among the 20 proteins carrying CBM1 modules, which increase the concentration of the enzymes at the surface of crystalline cellulose, 12 were linked to cellulose-degrading enzymes from (sub)families AA9, GH5_5, GH6, GH7 and GH131. Compared to other basidiomycete fungi, the transcriptome of *L. arvalis* also suggests a high potential for pectin, mannan and lignin breakdown (Figure 3), all of which are weak in *T. reesei*.

The presence of predicted pectinolytic enzymes of the PL1, PL3, PL4 and CE12 families is a feature shared with other soil basidiomycete fungi like *Agaricus bisporus* [16] and *Coprinopsis cinerea* [17]. The presence of one GH26-CBM35 enzyme together with three subfamily GH5_7 members, all putatively involved in mannan degradation, is another interesting feature of *L. arvalis*. Furthermore, 25 potential ligninolytic enzymes were confidently identified, including two laccases (AA1_1), 18 Glucose-Methanol-Choline oxidoreductases (AA3), three copper radical oxidases (AA5_1) and two benzoquinone reductases (AA6). Interestingly, *L. arvalis* contains two cellobiose dehydrogenases, contig07486 and contig08138, the latter lacking the iron reductase domain (AA8). *L. arvalis* also possesses at least five putative aryl-alcohol oxidases from subfamily AA3_2. Concerning subfamily AA5_1, all three gene models are similar to the genes encoding copper radical oxidases (CRO) in *Phanerochaete chrysosporium* (*cro2*, *cro5* and *cro6*). It is noteworthy that four modules of unknown function are fused to the N-terminus of one of the AA5_1 contig07281.

### Secretion of carbohydrate-active enzymes by *Laetisaria arvalis* is substrate-dependent

We analyzed the lignocellulose-degrading enzymes secreted by *L. arvalis* grown on different carbon sources (maltose, maize bran (MB), Avicel™ (AVI), WS and WS-R). Qualitative analysis of the electrophoretic profiles of the secretomes induced by the different carbon sources showed reliable reproducibility between biological duplicates (Additional file 1: Figure S5). Therefore, biological duplicates were pooled together for the rest of the study.

**Figure 4:**
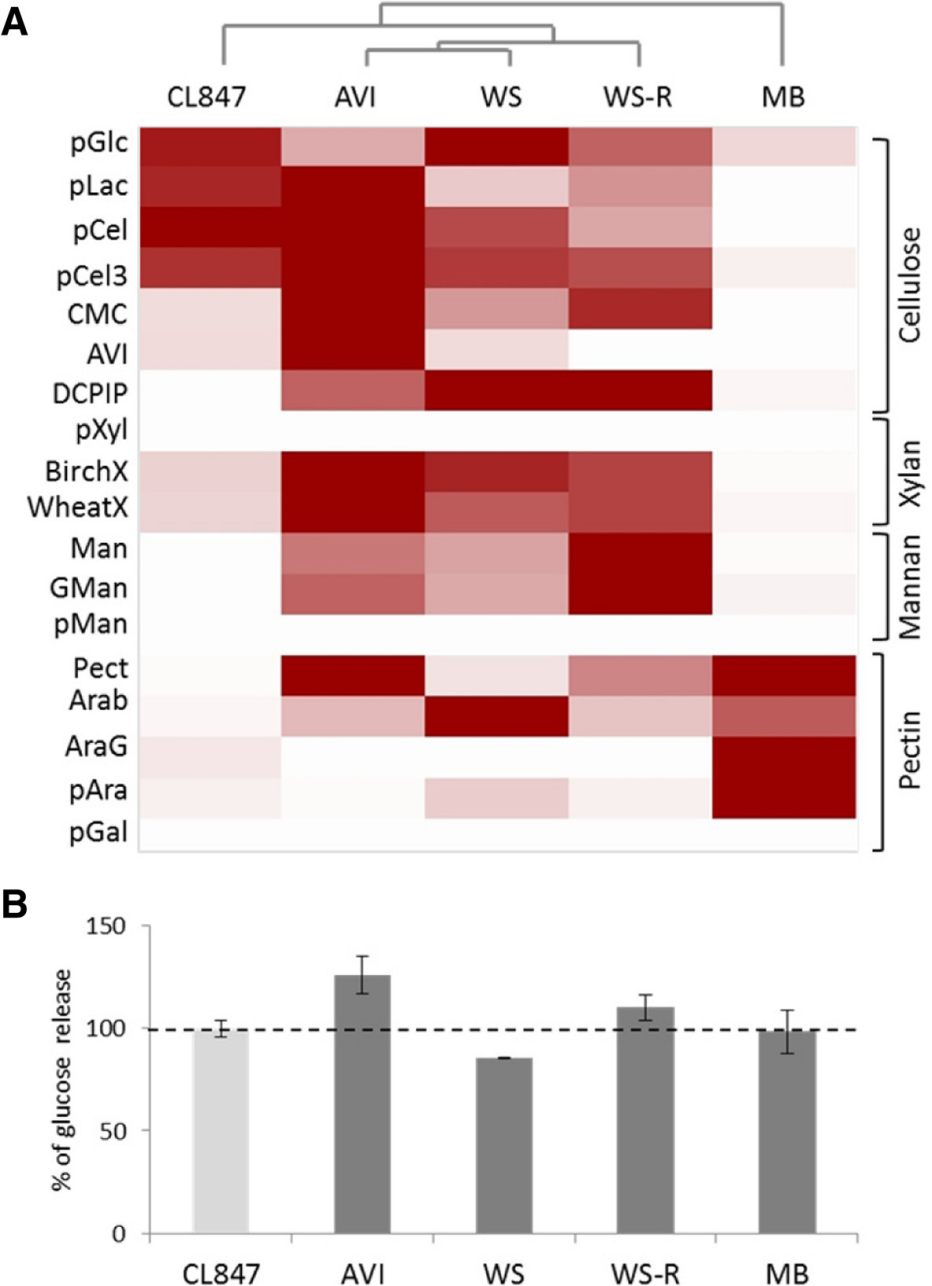
**Activity profiling of** ***Laetisaria arvalis*** **CAZymes and their potential to improve the conversion of pretreated wheat straw. A**: Clustering of the activities related to lignocellulose degradation in *L. arvalis* secretomes at day 10. The degree of activity of *L. arvalis* and *Trichoderma reesei* CL847 secretomes on the respective substrates is represented by a color scale with different strengths of red. The enzymatic activities of *L. arvalis* secretomes produced during growth on Avicel (AVI), wheat straw (WS), wheat straw residue following traditional saccharification (WS-R) and maize bran (MB) were determined on a library of substrates, pGlc, para-nitrophenol-β-D-glucose; pLac, para-nitrophenyl-β-D-lactose; pCel, para-nitrophenyl-β-D-cellobiose; pCel3, 2-chloro-4-nitrophenyl-β-D-cellotrioside; CMC, carboxymethyl cellulose; AVI, Avicel; DCPIP, 2,6-dichloro-phenol-indophenol; pXyl, para-nitrophenyl-β-D-xylose; BirchX, Birchwood xylan; WheatX, low viscosity wheat arabinoxylan; Man, mannan; GMan, galactomannan; pMan, para-nitrophenyl-β-D-mannose; Pect, pectin; Arab, arabinan; AraG, arabinogalactan; pAra, para-nitrophenyl-a-L-arabinose; pGal, para-nitrophenyl-β-D-galactose. The figure was edited using Multiexperiment Viewer [18]. **B**: Contribution of *L. arvalis* secretomes to the saccharification of steam-exploded WS. Biomass hydrolysis of was performed with fungal secretomes in combination with the *T. reesei* CL847 cocktail. The glucose released was quantified at the saccharification plateau as 30 μg of CL847 representing 100% sugar-releasing activity [19]. Values are means of six independent measures.

The activities of cellulases and hemicellulases were measured in each secretome and compared to those of the industrial enzymatic cocktail of *T. reesei* CL847. The highest endoglucanase (EG), cellobiohydrolase (CBH) and xylanase activities were measured in the AVI-induced condition while the highest β-glucosidase, mannanase and pectinase activities were measured in the WS-, WS-R- and MB-induced conditions, respectively (Figure 4A). Strikingly, the *L. arvalis* cellulase activity in the AVI-induced secretome was up to 7.5 times higher than that of the industrial enzymatic cocktail obtained from *T. reesei* strain CL847, at the same protein concentration (Additional file 1: Table S2).

We further analyzed the ability of *L. arvalis* secretomes to improve the saccharification of pretreated WS by the *T. reesei* enzyme mixture. Glucose release from steam-exploded WS was quantified at the saccharification plateau using each *L. arvalis* secretome in combination with the *T. reesei* CL847 enzymatic cocktail supplemented with β-glucosidase. Of the four secretomes tested, the AVI-induced secretome stood out from the others as it improved the glucose release by 26% (Figure 4B).

To determine the composition of the secretomes of *L. arvalis* induced with maltose, MB, AVI, WS and WS-R, we performed liquid chromatography-tandem mass spectrometry (LC-MS/MS) on secreted soluble proteins of 10-day-old cultures (Additional file 3: Data S2). Overall, 171 proteins were confidently identified by mass-matching against the proteins predicted from the *L. arvalis* transcriptome.

The secretomes from the five culture conditions differed in size from 22 proteins detected in the maltose condition to up to 129 identified in the WS condition. The ratio of GH, AA, PL and CE proteins was fairly similar between MB, AVI, WS and WS-R conditions and 12 core proteins were common to all the secretomes (Additional file 1: Figure S6), including two putative CBHs (GH7-CBM1, contig04291 and contig08217) and one putative CRO (AA5_1, contig07281) (Additional file 4: Data S3). Only two proteins were specific to the AVI condition, while 13, 29 and 10 proteins were specific to the MB, WS and WS-R conditions, respectively (Additional file 1: Figure S6, Additional file 4: Data S3). It is interesting to note that: (1) all five GH7 proteins (predicted to be CBHs) were identified together with the AA8-AA3_1/cellobiose dehydrogenase (CDH) (contig07486) in all of the inducing conditions except maltose, (2) the two proteins specific to the AVI condition were of unknown function (contig05465 and contig05873), (3) among the 12 proteins common to AVI, WS and WS-R, seven harbored a CBM1 module and (4) the putative laccase (AA1_1, contig08467) was identified exclusively in the WS condition and the AA5 CRO (contig04066) and AA5 glyoxal oxidase (contig04067) only found in the WS-R condition. The activity profiling and the proteomic analyses of the AVI secretome both highlighted enrichment in cellulose-degrading enzymes. Interestingly, out of all the secretomes we have explored, the AVI secretome exhibited the most enriched set of CAZymes (over 90% of the total number of identified peptides).

### The secretion of oxidases and hydrolases active on cellulose is time-regulated

**Figure 5:**
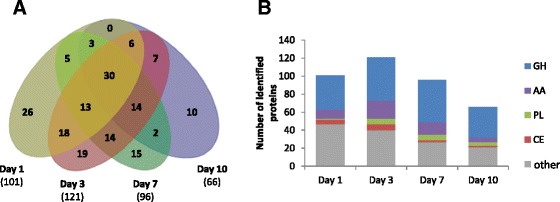
**Time-course secretion of proteins upon growth on Avicel™. A**: Venn diagram of the number of proteins detected and confidently identified by LC-MS/MS in the secretomes of *Laetisaria arvalis* at days 1, 3, 7 and 10 (from left to right). **B**: Distribution profile of CAZymes in secretomes over time. Bar size indicates the number of identified secreted proteins by class (GH, AA, PL, CE and other). AA, Auxiliary activity enzyme; CE, Carbohydrate esterases; GH, Glycoside hydrolase; LC-MS/MS, Liquid chromatography-tandem mass spectrometry; PL, Polysaccharide lyases.

**Figure 6:**
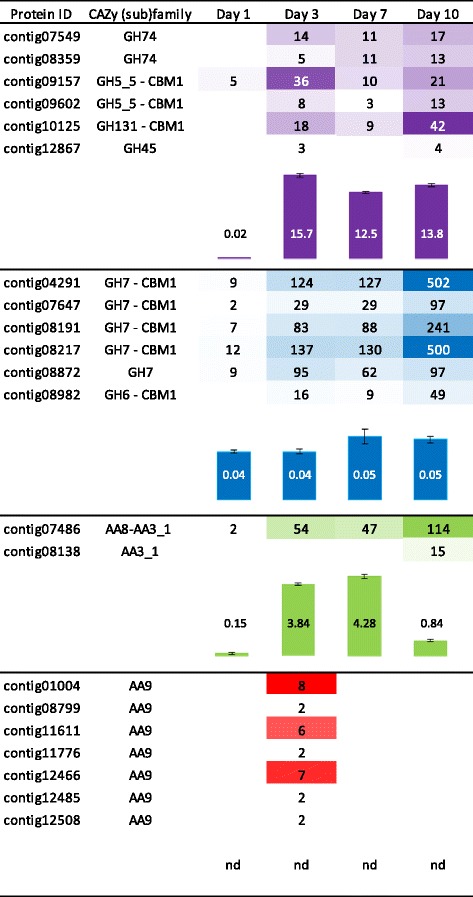
**Time-course abundance of cellulose-acting enzymes in secretomes during growth on Avicel™.** Abundance of cellulose-acting enzymes was determined based on the number of peptides unambiguously identified by LC-MS/MS, and the activities of endoglucanase (EG), cellobiohydrolase (CBH) and cellobiose dehydrogenase (CDH) were assayed using 2-chloro-4-nitrophenyl-β-D-cellotrioside (pCel3), para-nitrophenyl-β-D-lactose (pLac) and 2,6-dichloro-phenol-indophenol (DCPIP), respectively. For each putative enzyme, the CAZy module (sub)family is indicated. The contigs listed represent all detected secretomes members with putative EG, CBH, CDH and LPMOs activities. Peptides abundance is represented by a color scale with different strengths of purple, blue, green and red for EG, CBH, CDH and LPMOs, respectively. Activities are represented by bars (purple, blue, green and red for EG, CBH, CDH activities, respectively) and values indicated are expressed in U per mg of total proteins and are representative of triplicate measures. Error bars indicate standard errors of the mean. nd, not determined (as there is still no quantitative activity assay available for LPMOs).

As the AVI secretome significantly boosted the *T. reesei* enzymatic cocktail for WS conversion, we further studied how the temporal secretion of cellulose-acting enzymes is orchestrated in this growth medium. Over the course of 10 days, the amount of secreted soluble proteins increased with 2, 21, 32 and 58 mg of total protein per litre on days 1, 3, 7 and 10, respectively. LC-MS/MS analysis revealed differences in the composition of the secretomes and a maximum protein diversity at day 3 with 121 proteins identified (Figure 5). A core of 30 proteins was common to all time points, including five putative GH7/CBHs (contig04291, contig07647, contig08191, contig08217 and contig08872) and one putative AA8-AA3_1/CDH (contig07486) (Additional file 4: Data S3). This set was completed from day 3 on by putative cellulose-acting from (sub)families GH5_5, GH6, GH45, GH74 and GH131. Overall EG, CBH and CDH activities were assayed for the AVI secretomes from day 1 to day 10 (Figure 6). EG and CBH activities remained high and stable over time (from day 3 to 10), which is consistent with the identification of cellulose-active enzymes in the secretomes, whereas CDH activity peaked at days 3 and 7. The drop in CDH activity at day 10 was not concordant with the time-course of CDH secretion, estimated based on the number of peptides identified by LC-MS/MS. This points to a possible proteolytic cleavage separating the flavin AA3_1 module from the heme AA8 module, thus preventing the electron transfer required for optimal CDH activity [20].

Strikingly, LC-MS/MS analysis of the AVI secretome at day 3 showed the secretion of seven different putative LPMOs (contig01004, contig08799, contig11611, contig11776, contig12466, contig12485 and contig12508), all belonging to family AA9 (Figure 6). MS spectra supported the methylation of the strictly-conserved N-terminal histidine residue in two LPMOs (contig11611, contig12466). The N-terminal peptides of the other five LPMOs were not detected (Additional file 1: Table S3). This observation is coherent with the methyl group attached to atom Nε2 of the histidine contributing to copper-ion coordination found in crystal structures [21,22], although the biochemical role of this quite rare post-translational modification in the oxidative degradation of cellulose remains uncertain [23]. The disappearance of LPMOs at day 7 coincided with the emergence of several proteases (Additional file 3: Data S2).

**Figure 7:**
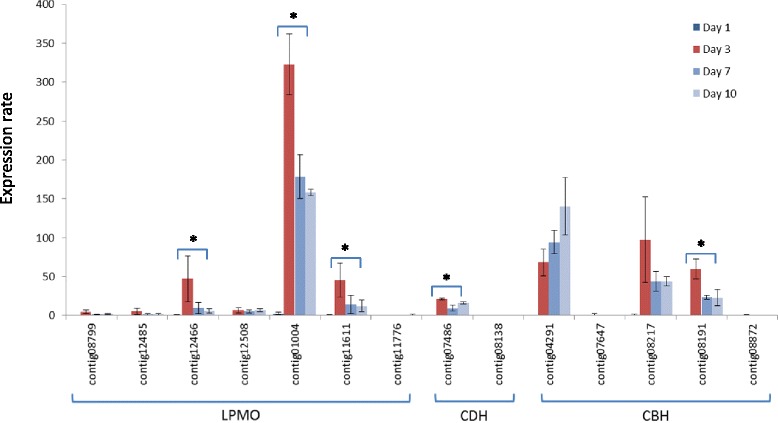
**Expression levels of LPMO-, CDH- and CBH-encoding genes determined by RT-qPCR.** Normalized expression rates of genes encoding five GH7 CBHs (contig04291, contig07647, contig08191, contig08217 and contig08872), seven AA9 LPMOs (contig01004, contig08799, contig11611, contig11776, contig12466, contig12485 and contig12508) and two CDHs (contig07486 and contig08138) relative to the *actin-1* transcript (contig09769) were quantified in mycelia grown for one, three, seven and 10 days in the Avicel™ condition. Error bars indicate the standard deviation for three biological replicates. * indicates significant difference (a *P*-value of <0.05 in an equal variance, one-tailed Student’s t-test) between day 3 and day 7 transcript levels. CBH, Cellobiohydrolase; CDH, Cellobiose dehydrogenase; LPMO, Lytic polysaccharide monooxygenase.

To further investigate the regulation of the expression of genes encoding *L. arvalis* cellulose-degrading enzymes, we examined the relationship between protein abundance and transcript levels for the seven LPMOs, the two CDHs and the five GH7/CBHs, identified in the AVI condition (Figure 6). The patterns of expression of these genes relative to the *actin-1* transcripts between day 1 and day 10 were investigated by reverse transcription quantitative polymerase chain reaction (RT-qPCR) (Figure 7). Overall, we detected expression of all of the transcripts. The relative abundance of transcripts was in good agreement with the abundance of peptides identified by LC-MS/MS (Figures 6 and 7). The transcripts displaying the highest induction level at day 3 were contig01004, contig11611 and contig12508 for LPMOs, contig07486 for CDH and contig08191 for CBH. In agreement with proteomic analyses of the secretomes, the expression of the gene encoding the CDH lacking the flavin domain (contig08138) was not induced.

## Discussion

This paper reports and describes the remarkable natural cellulose-degrading ability of the basidiomycete *L. arvalis*. The complete deconstruction of filter paper strips within 10 days, and the rapid growth of its mycelium on recalcitrant biomass as the sole carbon source prompted us to further investigate the cellulolytic machinery of this fungus through a combination of biochemical, transcriptomic and proteomic analyses.

*L. arvalis* is a soil-inhabiting basidiomycete fungus classified in the Corticiales order recently established [24]. This group of fungi includes mainly wood-rotting fungi and parasites of grasses (*Laetisaria fuciformis* and *Erythricium salmonicolor*) and lichens (*Marchandiomyces* and *Marchandiobasidium*). In this study, the sequencing and *de novo* assembly of the global transcriptome of *L. arvalis* CIRM-BRFM514 revealed that only 19% of the transcripts were related to genes from the Corticiales fungus *Punctularia strigosozonata*, a widespread resupinate fungus that causes white-rot decay on hardwood [8]. The transcriptome sequencing of *L. arvalis* provides important data for a previously underexplored basidiomycete fungus that will help to characterize the Corticiales order. The global sequencing of fungal transcriptomes is thus a pertinent approach to investigate fungal strains distantly related to reference fungal genomes and a promising alternative to genomic sequencing of basidiomycetes displaying heterokaryotic cells.

Different types of decay patterns observed in fungi have been shown to be related to gene families encoding CAZymes (mainly GH and AA families) present in their genome [8]. GH-encoding genes, particularly those acting on crystalline cellulose, are omnipresent in white-rot genomes. For instance, genes encoding family GH6 and GH7 enzymes, which include EGs and CBHs involved in the breakdown of crystalline cellulose [25], are present in all white-rot lineages but absent in most brown-rots. Similar patterns of enrichment in white-rot genomes are also shown for genes encoding AA9/LPMOs and CBM1 module-containing proteins [8]. In this regard, *L. arvalis* resembles white-rot species, with a complete set of CAZyme families related to cellulose degradation, including abundant GH7, AA9 and CBM1 gene copies. On the other hand, *L. arvalis* contains a number of laccases and CRO but no family AA2 class II peroxidase, suggesting that this litter-rot fungus departs from the classical white-rot basidiomycete fungi [26]. This difference is accompanied by the presence of a very large set of enzymes putatively involved in pectin degradation (GH28, GH43, GH51, GH54, GH88, GH105, PL1, PL3 and PL4). *L. arvalis* also displays a single GH26 and the largest set of GH5_7, most likely devoted to β-mannan degradation, compared to a collection of white-, brown- and litter-rot basidiomycetes (Figure 3). The unique *L. arvalis* enzyme repertoire in the fungi taxa sequenced to date highlights the adaptation of this litter-rot fungus to lignocellulose breakdown. Although the exact substrate range of *L. arvalis* is not known, its extensive and diverse spectrum of plant polysaccharide-breakdown enzymes suggests that it could grow on a wide range of plant cell wall debris encountered in soil litter.

Fungal breakdown of plant biomass requires the secretion of many different enzymes, which is finely regulated in response to the type and complexity of the encountered plant material [13,27–29]. Here, the comparative analysis of *L. arvalis* secretomes induced by diverse lignocellulosic substrates revealed striking differences in the secreted enzymatic pools that were in good agreement with the main sugar-cleaving activities measured. While only a few proteins were secreted in response to maltose, crystalline cellulose (AVI) induced the secretion of a very complete set of cellulolytic enzymes whose combination has significant relevance for improved conversion of biomass. Temporal analysis of the AVI secretomes revealed a core of five GH7/CBHs and one AA8-AA3_1/CDH among the most abundant secreted proteins over time.

It is well established that the hydrolytic efficiency of cellulose by *T. reesei* is mainly reliant on two CBHs from families GH6 and GH7 (TrCel6A and TrCel7A, respectively) that are produced in large amounts and have been shown to act processively from the non-reducing end and from the reducing end of cellulose chains, respectively [30]. Each of the five GH7/CBHs of *L. arvalis* shares less than 65% sequence identity with TrCel7A, and the closest GH7 members characterized from the white-rot fungi *P. chrysosporium* and *Irpex lacteus* display less than 70% sequence identity. The five GH7/CBHs of *L. arvalis* may thus differ in terms of specific activity and processivity, leading to improved degradation of crystalline cellulose and/or hydrolysis of amorphous cellulose, as demonstrated in a comparative analysis of three fungal CBHs from *T. reesei* and *P. chrysosporium* [31]. Moreover, the core set of *L. arvalis* GH7/CBHs is completed by several other predicted cellulose-degrading enzymes from (sub)families GH5_5, GH6, GH45, GH74 and GH131, including several predicted EGs that could favor the action of CBHs by creating chain ends. This enzyme mixture could be seen as the *L. arvalis* ‘minimal enzyme cocktail’ (a concept described elsewhere [32]), that is, the optimal combination of the best-performing key components for efficient cellulose deconstruction.

A variety of white-rot, brown-rot and other fungi have been reported to secrete CDH when grown on cellulose [33]. CDHs have been proposed to play an important role in biomass breakdown. It has been suggested that they could enhance cellulase activity by relieving product inhibition through the oxidation of cellobiose to cellobionic acid [34], and more recent work has demonstrated that CDH was able to act synergistically with AA9 in cellulose breakdown by coupling cellobiose oxidation to the reductive activation of AA9 proteins [35,36]. Analysis of fungal genomes has revealed that basidiomycetes possess an average number of approximately 0.6 AA8-AA3_1 (CDH), whereas AA9 members are more uniformly distributed [37]. Fungal transcriptome and secretome studies have shown that AA9 LPMOs isoforms are specifically secreted during degradation of lignocellulosic substrates, but the relevance of this biological regulation is still unknown [13,38–41]. Although the global *L. arvalis* transcriptome accounts for 16 LPMOs, we observed in the AVI condition a spike of seven AA9/LPMOs secreted at an early stage of growth (day 3). On the contrary, no LPMO was found at day 10 in the secretomes in our growth conditions (maltose, MB, AVI, WS and WS-R).

Induction of the expression of the five GH7/CBHs, the AA8-AA3_1/CDH and three AA9/LPMOs (contig01004, contig11611 and contig12466) at day 3 on AVI suggests that the efficiency of *L. arvalis* in the breakdown of cellulose could be driven by the sequential secretion of oxidative and hydrolytic enzymes. Indeed, LPMOs and CDH could play a major role at the early stages of cellulose deconstruction by creating oxidative breaks on cellulose fibers that could subsequently facilitate the action of CBHs and EGs that accumulate in the secretome. It therefore is likely that the oxidative processes catalyzed by *L. arvalis* LPMOs play a major role in initiating and potentiating biomass conversion. Interestingly, a recent study demonstrates that LPMOs could also cleave hemicelluloses [42], which broaden oxidative processes to non-cellulosic polysaccharides from plants. The importance of the early stages of biomass deconstruction is further highlighted by the secretion of a protein with a predicted CBM1 (contig12628) only at day 3 in the AVI condition. Although the precise function of this protein cannot be predicted, the presence of a cellulose-binding CBM1 module and the fact that it was secreted together with AA9/LPMOs points to a potential role in biomass degradation.

## Conclusions

The present study illustrates adaptation of a litter-rot fungus to the rapid breakdown of recalcitrant plant biomass, further demonstrating that fungal saprotrophs may degrade lignocellulose using very diverse strategies. The extraordinary cellulolytic capabilities of this soil-inhabiting basidiomycete fungus result from the rapid, selective and successive secretion of oxidative and hydrolytic enzymes. These enzymes expressed at critical times during biomass degradation may inspire the design of improved enzyme cocktails for the conversion of plant cell wall resources into fermentable sugars.

## Materials and Methods

### Biomass substrates

WS (*Triticum aestivum*, Apache, Reims, France) was prepared as described elsewhere [43]. WS saccharification residues (WS-R) were obtained from Dr Antoine Margeot (Institut Français du Pétrole - Energies Nouvelles, Rueil-Malmaison, France). They were generated from steam-exploded WS under acidic conditions followed by saccharification with a *T. reesei* CL847 enzyme cocktail using standard saccharification procedures. WS-R were extensively washed with deionized water and dried at room temperature.

### Fungal strains

The *L. arvalis* strain used in this study was originally isolated in Nebraska, United States [43] and was obtained from the CBS-KNAW culture collection (Fungal diversity centre, Utrecht, The Netherlands), accession number [CBS131.82]. It was verified by Internal Transcribed Spacer (ITS) sequencing and maintained on malt agar slants, using MA2 (malt extract at 2% w/v) medium, in the fungal culture collection of the International Centre of Microbial Resources (CIRM-CF) at the French National Institute for Agricultural research (INRA; Marseille, France) under accession number [CIRM-BRFM514].

The wild-strain *T. reesei* QM6a widely used for cellulolytic enzyme production (RUT-C30, QM9414) [3], was maintained on agar slants, using MYA2 (malt extract at 2%, w/v and yeast extract 0.1%, w/v) as medium.

### Growth ability on biomass and cellulose as sole carbon source

To assay the ability of *L. arvalis* to grow on plant biomass, 5 g of WS or WS-R were autoclaved (30 minutes at 110°C) before addition of 15 mL minimum medium (12 g/L NaNO_3_, 2 g/L KH_2_PO_4_, 1 g/L KCl and 1 g/L MgSO_4_) and 200 μL of Vischniac trace elements [44]. To test growth ability on cellulose, *L. arvalis* and *T. reesei* QM6a were grown on minimum medium (10.72 g K_2_HPO_4_; 5.24 g KH_2_PO_4_; 2 g (NH_4_)2SO_4_; 0.5 mL iron solution (1 mg of FeSO_4_ dissolved in 1 mL in 0.01 M HCl); 1 mL 1 M MgSO_4_ solution and 5 mL of trace elements solution (SPV-4)) [45], containing one strip (10 × 1 cm) of Whatman #1 filter paper (Sigma-Aldrich, Saint-Quentin Fallavier, France) as the sole carbon source. The sugar content (w/w) of WS and WS-R determined using the alditol acetate method [46] is reported in Additional file 1: Table S1. Cultures were inoculated with mycelial fragments from five fungal disks (4 mm diameter) crushed in 1 mL minimum medium, using a FastPrep-24 system (MP Biomedicals, Illkirch, France) set to 40 m/s^−1^ for 60 seconds. Liquid cultures were incubated at 30°C with shaking at 160 rpm.

### RNA extraction, cDNA library construction and sequencing

*L. arvalis* was grown in 250 mL baffled Erlenmeyer flasks with 50 mL liquid medium containing 2.5 g/L^−1^ of maltose as a starter, 15 g/L^−1^ (based on dry matter) of AVI or WS for 1, 3, 5, 7 and 10 days. For each condition, three Erlenmeyer flasks were utilized. The cultures were centrifuged at 4,000 rpm for 5 minutes, in order to separate large biomass particles from the lighter mycelium. The supernatant was discarded and the mycelium carefully collected with a spatula. The mycelium was ground in liquid nitrogen using a 6770 Freezer/Mill (SPEX SamplePrep LLC, Metuchen, United States). Total RNAs were extracted from 100 mg ground mycelium with 1 mL TRIzol reagent (Ambion, Life Technologies, Saint-Aubin, France) according to the manufacturer’s instructions. Total RNA was precipitated with LiCl and resuspended in 25 μL diethylpyrocarbonate (DEPC)-treated water. RNA quantity was determined using NanoDrop 2000 (Thermo Fisher Scientific, Illkirch, France). RNA samples from all the sampling days were pooled and quality was assessed by capillary electrophoresis using a BioAnalyzer (Agilent). The RNA sample was sent to GATC Biotech (Konstanz, Germany) for subsequent library preparation as described elsewhere [47].

### Transcriptome assembly and functional annotation

After quality check and trimming of the adapter sequences, the sequence reads were assembled using Roche GS *de novo* Assembler (Newbler software) with default parameters for cDNA. Functional annotation of the resulting contigs was performed using the Blast2GO software package [48]. BlastX was performed against the NCBI nr database with default parameters except for the minimum E-value set at 10^−6^.

### Functional annotation of CAZymes

Annotation of the set of CAZymes was performed by comparing the predicted proteins to the CAZy database [14]. As CAZy annotation is based on the recognition of individual catalytic modules or CBMs, it is possible to assess whether an assembled contig encoding a CAZyme covers the full length of the identified modules. Only the contigs with more than 80% overlap with a reference sequence were validated as belonging to one or more of the CAZy families, depending on their modular composition. Comparison of the CAZy set of various fungi was performed by double hierarchical clustering using the Ginkgo Multivariate Analysis System (Singleton Labs, Kaunas, Lithuania).

### Preparation of *Laetisaria arvalis* secretomes

Based on previous studies [12,13], fungal cultures were grown in 250 mL baffled Erlenmeyer flasks with 100 mL medium containing 2.5 g/L^−1^ of maltose as a starter, 15 g/L^−1^ (based on dry matter) of an autoclaved MB fraction (provided by ARD, Pomacle, France), Avicel® (Avicel PH-101, Sigma-Aldrich), WS and WS-R as a carbon source, 1.842 g/L^−1^ of diammonium tartrate as a nitrogen source, 0.5 g/L^−1^ yeast extract, 0.2 g/L^−1^ KH_2_PO_4_, 0.0132 g/L^−1^ CaCl_2_/2H_2_O and 0.5 g/L^−1^ MgSO_4_/7H_2_O. In parallel, a reference culture was made with 20 g/L^−1^ of maltose as a carbon source. Cultures were incubated in the dark at 30°C with shaking at 120 rpm. The cultures were stopped 10 days after inoculation in all the inducing conditions, and the culture broths (secretomes) were filtered (using 0.2 μm polyethersulfone membrane, Vivaspin, Sartorius), diafiltered with 50 mM acetate solution buffer pH 5.2, concentrated (Vivaspin with a 10 kDa cut-off polyethersulfone membrane, Sartorius) and then stored at −20°C until use.

The total amount of proteins was assessed using Bradford assays (Bio-Rad Protein Assay Dye Reagent Concentrate, Ivry, France) with a BSA standard that ranged from 0.2 to 1 mg/mL^−1^. Gel electrophoresis and carboxymethyl cellulose zymograms were performed as described elsewhere [49].

### Enzyme activity measurement

*p*-nitrophenol-based chromogenic substrates and complex polysaccharide substrates were used to assay the enzymatic activities of the fungal secretomes as described elsewhere [13]. EG activity was also assayed using 2-chloro-4-nitrophenyl-β-D-cellotrioside (pCel3, Megazyme, Wicklow, Ireland) following the manufacturer’s instructions. CDH activity was measured using 2,6-dichloro-phenol-indophenol (DCPIP) as described elsewhere [49].

### Saccharification assays

The concentrated secretomes were tested for their ability to hydrolyze steam-exploded WS that was dispensed into 96-well plates by the Tecan Genesis Evo 200 robot (Tecan, Lyon, France) as described elsewhere [19]. To quantify the sugars released at the saccharification plateau (24 hours), 17, 27, 12.5 and 16 μg of total proteins (for AVI, WS, WS-R and MB, respectively) were added to the substrate plate along with 30 μg of the *T. reesei* CL847 enzymatic cocktail (enzyme composition described elsewhere [13]). Glucose release was analyzed using a glucose RTU™ kit (Biomérieux, Marcy l’Etoile, France) following the manufacturer’s instructions. All control experiments were performed according to Navarro *et al*. [19] and reactions were performed independently at least six times.

### Proteomic analysis of secretomes

Short SDS-PAGE runs (pre-casted 4 to 12% Bis-Tris Mini Gels, Invitrogen, France) were performed, allowing proteins diafiltered from secretomes (15 μg) to migrate on a 0.5 cm length, and gels were stained with Coomassie blue (BioRad, Marnes-la-Coquette, France). Each one-dimensional electrophoresis lane was cut into two slices of gel (2 mm in width) and protein identification was performed using PAPPSO (“Plate-forme d'Analyse Protéomique de Paris Sud-Ouest”) platform facilities. In-gel digestion was carried out according to a standard trypsinolysis protocol. Gel pieces were washed twice with 50% (v/v) acetonitrile (ACN), 25 mM NH_4_CO_3_ and incubated in the presence of 10 mM dithiothreitol (DTT) for 1 hour at 56°C. After cooling, the supernatant was removed and the samples were incubated with 55 mM iodoacetamide at room temperature in the dark. Gel plugs were washed with ACN and then dried in a vacuum speed concentrator. Digestion was performed for 8 hours at 37°C with 200 ng of modified trypsin (Promega, Charbonnières-les-Bains, France) dissolved in 25 mM NH_4_CO_3_. Tryptic peptides were first extracted with 50% (v/v) ACN, 0.5% (v/v) trifluoroacetic acid (TFA), and then with pure ACN. Peptide extracts were dried in a vacuum speed concentrator (Thermo Fisher Scientific, Villebon sur Yvette, France) and suspended in 25 μL of 2% (v/v) ACN, 0.05% (v/v) TFA and 0.08% (v/v) formic acid. HPLC was performed on a NanoLC-Ultra system (Eksigent, Les Ulis, France). Trypsic digestion products were first concentrated and desalted on a pre-column cartridge (PepMap 100 C_18_, 0.3 × 5 mm, Dionex, Thermo Fisher Scientific) with 0.1% HCOOH at 7.5 μL/min^−1^ for 3 minutes. The pre-column cartridge was connected to the separating column (C_18_, 0.075 × 0.15 mm, Biosphere Nanoseparations, Nieuwkoop, The Netherlands) and the peptides were eluted with a linear gradient from 5 to 35% ACN in 0.1% HCOOH for 40 minutes at 300 nL/min^−1^. On-line analysis of peptides was performed with a Q-exactive mass spectrometer (Thermo Fisher Scientific, United States), using a nanoelectrospray ion source. Ionization (1.8 kV ionization potential) was performed with a stainless steel emitter (30 μm inner diameter , Thermo Electron, Villebon sur Yvette, France). Peptide ions were analyzed using Xcalibur 2.1 (Thermo Scientific, Villebon sur Yvette, France with the following data-dependent acquisition steps: (step 1) full MS scan (mass-to-charge ratio (m/z) 400 to 1,400, resolution 70,000) and (step 2) MS/MS (normalized collision energy =30%, resolution 17,500). Step 2 was repeated for the eight major ions detected in step 1. Dynamic exclusion was set to 40 seconds. The raw mass data were first converted to mzXML format with the ReAdW software (SPC Proteomics Tools, Seattle, USA). Protein identification was performed querying MS/MS data against databases, together with an in-house contaminant database, using the X!Tandem software (X!Tandem Cyclone, Jouy en Josas, France)))] with the following parameters: one trypsin missed cleavage allowed, alkylation of cysteine and conditional oxidation of methionine, precursor and fragment ion set at 2 ppm and 0.005 Da, respectively. A refined search was added with similar parameters except that semi-tryptic peptides, possible N-term acetylation, and histidine mono- and di-methylations were searched. All peptides matched with an E-value lower than 0.05 were parsed with X!Tandem pipeline software. Proteins identified with at least two unique peptides and a log (E-value) lower than −2.6 were validated. A list of proteins and peptides with masses and log (E-value) is provided in Additional file 2.

### Expression analyses (real time PCR)

*L. arvalis* mycelium was collected after 1, 3, 7 and 10 days of growth in the AVI medium. Total RNAs were extracted, precipitated and quantified as described below. Single-stranded cDNAs were synthesized from 2 μg RNA and amplified using an i-Script cDNA Synthesis kit (Bio-Rad, Marnes-la-Coquette, France). First strand cDNAs were diluted to one fifth and used as template for real-time PCR using qPCR SsoADV SYBR Green Mix (Bio-Rad, Marnes-la-Coquette, France) on a CFX96 Touch Real-Time PCR Detection System (Bio-Rad), as follows: 95°C for 30 seconds and 40 cycles of 95°C for 5 seconds, 57°C for 5 seconds followed by a melting curve analysis. Standard curves were established with four serial dilutions of amplified cDNAs, ranging from one fifth to one in 50,000. Five *L. arvalis* housekeeping genes were tested for expression level variation after 1, 3, 7 and 10 days culture in our conditions (*actin-1* (contig09769), *actin-2* (contig00536), *alpha tubulin-1* (contig08662), *alpha tubulin-2* (contig08988) and *chitin synthase* (contig13814)). The *L. arvalis actin-1* gene was selected as the reference gene because its expression level was most stable throughout the culture (data not shown). Gene-specific primers were designed using the Primer 3 software [50] (Additional file 1: Table S4). The regression cycle (Cq) values were determined on technical duplicates for each of three independent biological samples at each time point. Calculations of the normalized expression rates were performed according to the ΔC_t_ method.

## Additional files

Additional file 1:
**Supplementary Figures (S1-S6) and Tables (S1-S4).**
Additional file 2: Data 1. Fasta file containing the 15,679 contigs. Additional file 3: Data 2. Excel table containing the list of the proteins detected and confidently identified in *L. arvalis* secretomes at day 10 (maltose, MB, AVI, WS and WS-R growth conditions) and from day 1 to day 10 (AVI growth condition). Additional file 4: Data 3. Table listing proteins relative to the Venn diagrams presented in Figure 5 and Additional file 1: Figure S6.

## Abbreviations

AA: Auxiliary activity enzyme
AVI: Avicel
CAZyme: Carbohydrate-active enzyme
CBH: Cellobiohydrolase
CBM: Carbohydrate-binding module
CDH: Cellobiose dehydrogenase
CE: Carbohydrate esterases
CIRM: International Centre of Microbial Resources
CRO: Copper radical oxidases
DEPC: diethylpyrocarbonate
DTT: dithiothreitol
EG: Endoglucanase
GH: Glycoside hydrolase
GO: Gene ontology
GT: Glycosyltransferase
LC-MS/MS: Liquid chromatography-tandem mass spectrometry
LPMO: Lytic polysaccharide monooxygenase
MB: Maize bran
PAPPSO: Plate-forme d'Analyse Protéomique de Paris Sud-Ouest
PL: Polysaccharide lyases
RT-qPCR: reverse transcription quantitative polymerase chain reaction
WS: Wheat straw
WS-R: WS residue
