# Supplementary material for: Fast solubilization of recalcitrant cellulosic biomass by the basidiomycete fungus Laetisaria arvalis involves successive secretion of oxidative and hydrolytic enzymes
Source: Biotechnol Biofuels. 2014 Oct 8;7:143. doi: 10.1186/s13068-014-0143-5 (PMC4197297; doi:10.1186/s13068-014-0143-5)
Supplement: Additional file 1: — Supplementary Figures (S1-S6) and Tables (S1-S4). [file 13068_2014_143_MOESM1_ESM.docx]

**Supplementary Figures**

**
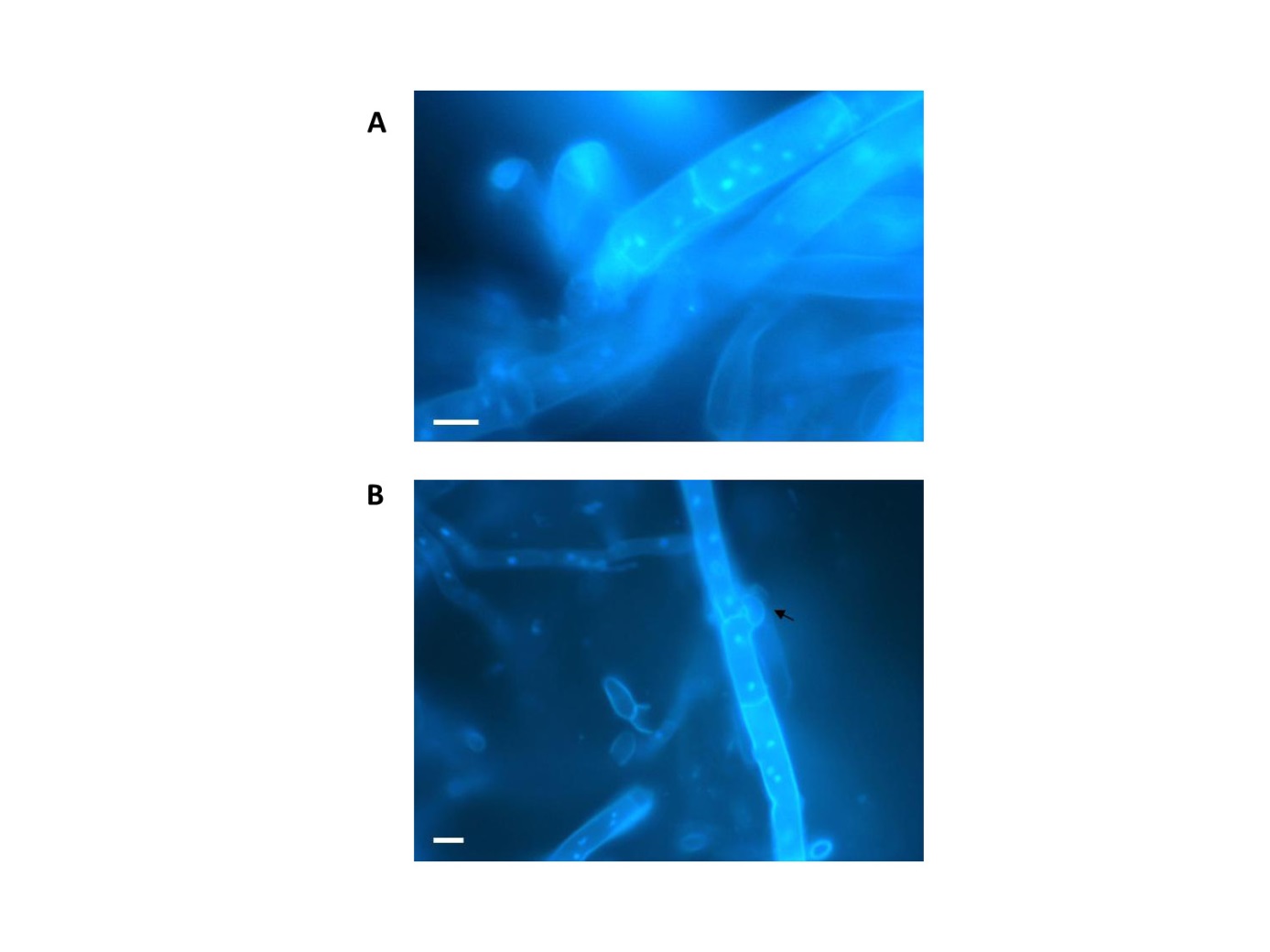
**

**Figure S1: *L. arvalis* hyphal system produced in agar medium, stained with DAPI and observed using fluorescence microscopy.** Septate and multinucleate hyphae (A); Characteristic clamp connection (arrow) of dikaryotic hyphae (B). The DAPI stained the cell nuclei. Bar = 10 mm.

**A**

| **Transcriptome assembly** |  |
| --- | --- |
| No. of 454-reads | 367 307 |
| No. of clusterized reads | 282 749 |
| No. of reads assembled into contigs | 240 696 |
| No. of contigs assembled | 15 679 |
| No. of singletons | 31 837 |
| Average contig length (bases) | 1 185 |
| Largest contig length (bases) | 7 730 |

**B**

**Figure S2: Assembly process statistics (A) and size distribution of the contig generated (B).**

**B**

**A**

**Figure S3: Blast2GO functional annotation statistics.** Distribution of the Sequence similarity values for the BlastX hits (A) and Taxonomic origin at the specie level of the BlastX Top hits (B).

**
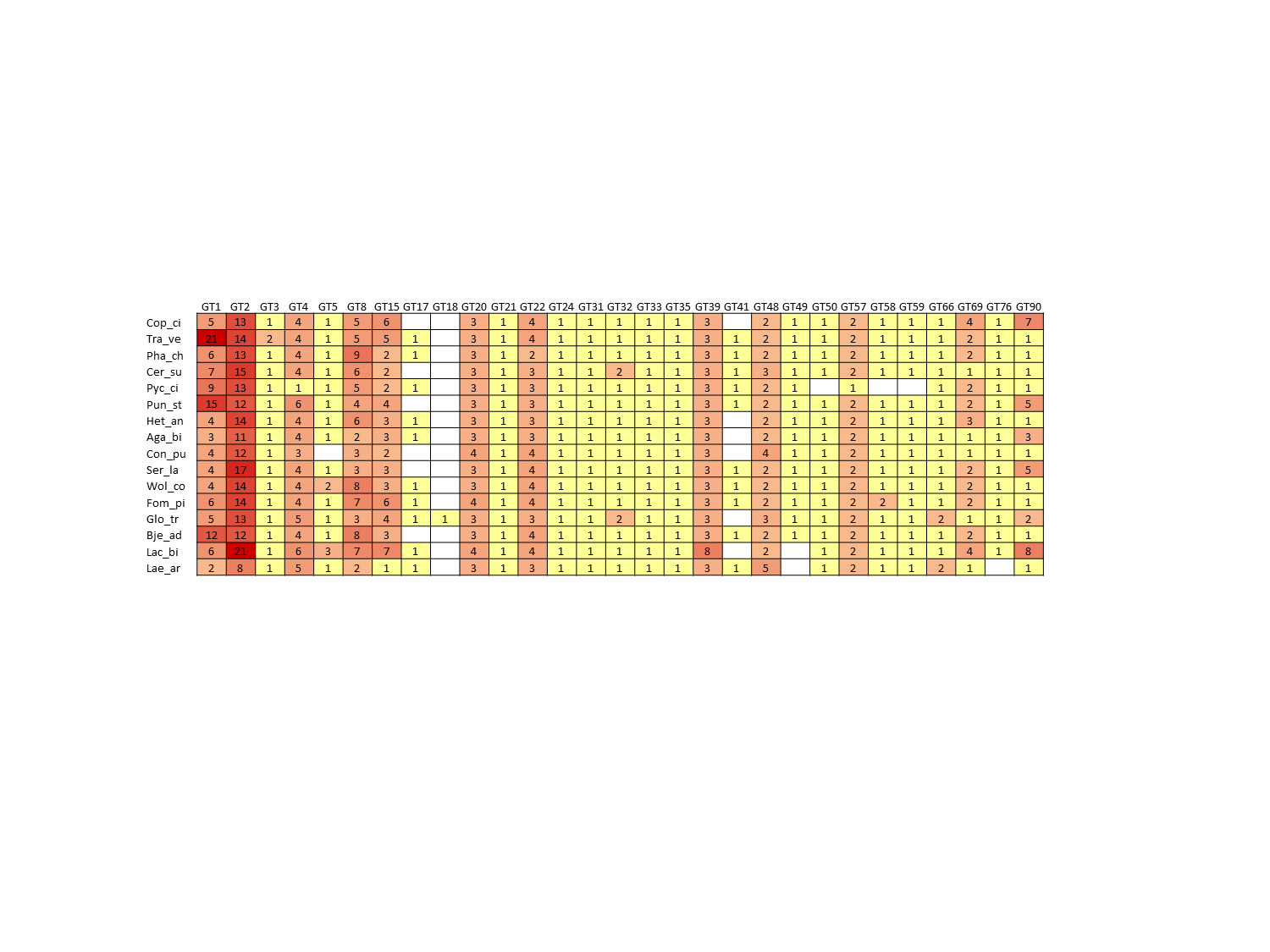
**

**Figure S4: Comparison of *L. arvalis* Glycosyl Transferases (GTs) to other fungi.** The abundance of the number of genes encoding GTs is represented by a colour scale. The fungal genomes analyzed are Cop_ci, *Coprinopsis cinerea*; Tra_ve, *Trametes versicolor*; Pha_ch, *Phanerochaete chrysosporium*; Cer_su, *Ceriporiopsis subvermispora*; Pyc_ci, *Pycnoporus cinnabarinus*; Pun_st, *Punctularia strigosozonata*; Het_an, *Heterobasidion annosum;* Aga_bi, *Agaricus bisporus var. burnettii*; Con_pu, *Coniophora puteana*; Ser_la, *Serpula lacrymans*; Wol_co, *Wolfiporia cocos*; Fom_pi, *Fomitopsis pinicola*; Bje_ad, *Bjerkandera adusta*; Lac_bi, *Laccaria bicolor*; Glo_tr, *Gloeophyllum trabeum*; Lae_ar, *Laetisaria arvalis*.

**
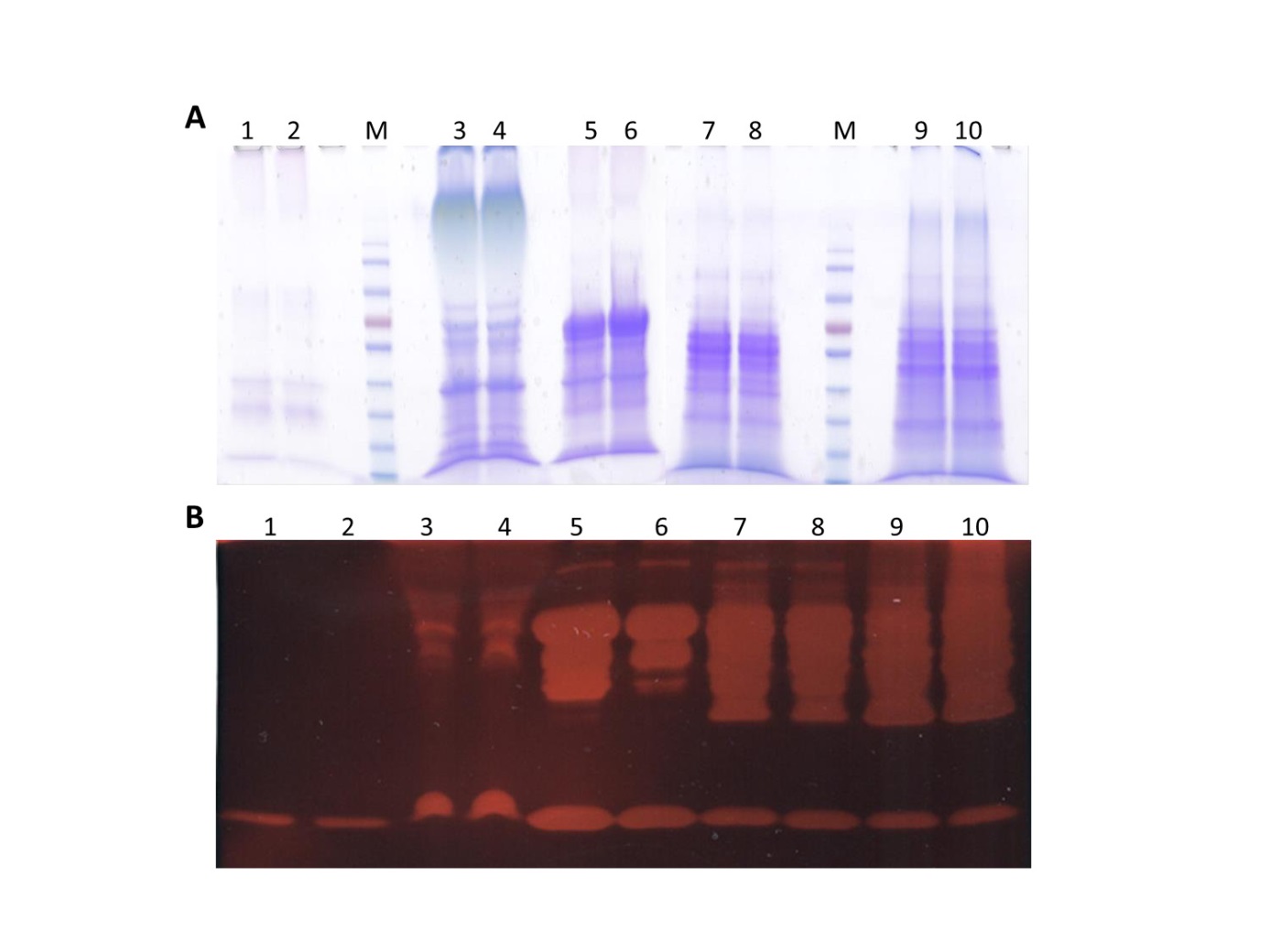
**

**Figure S5: Electrophoretic analyses of *L. arvalis* secretomes.** SDS-PAGE (A) and CMC-zymogram (B). Lanes: 1-2, maltose; 3-4, MB; 5-6, AVI; 7-8, WS; 9-10, WS-R. M, pre-stained molecular mass markers.

**
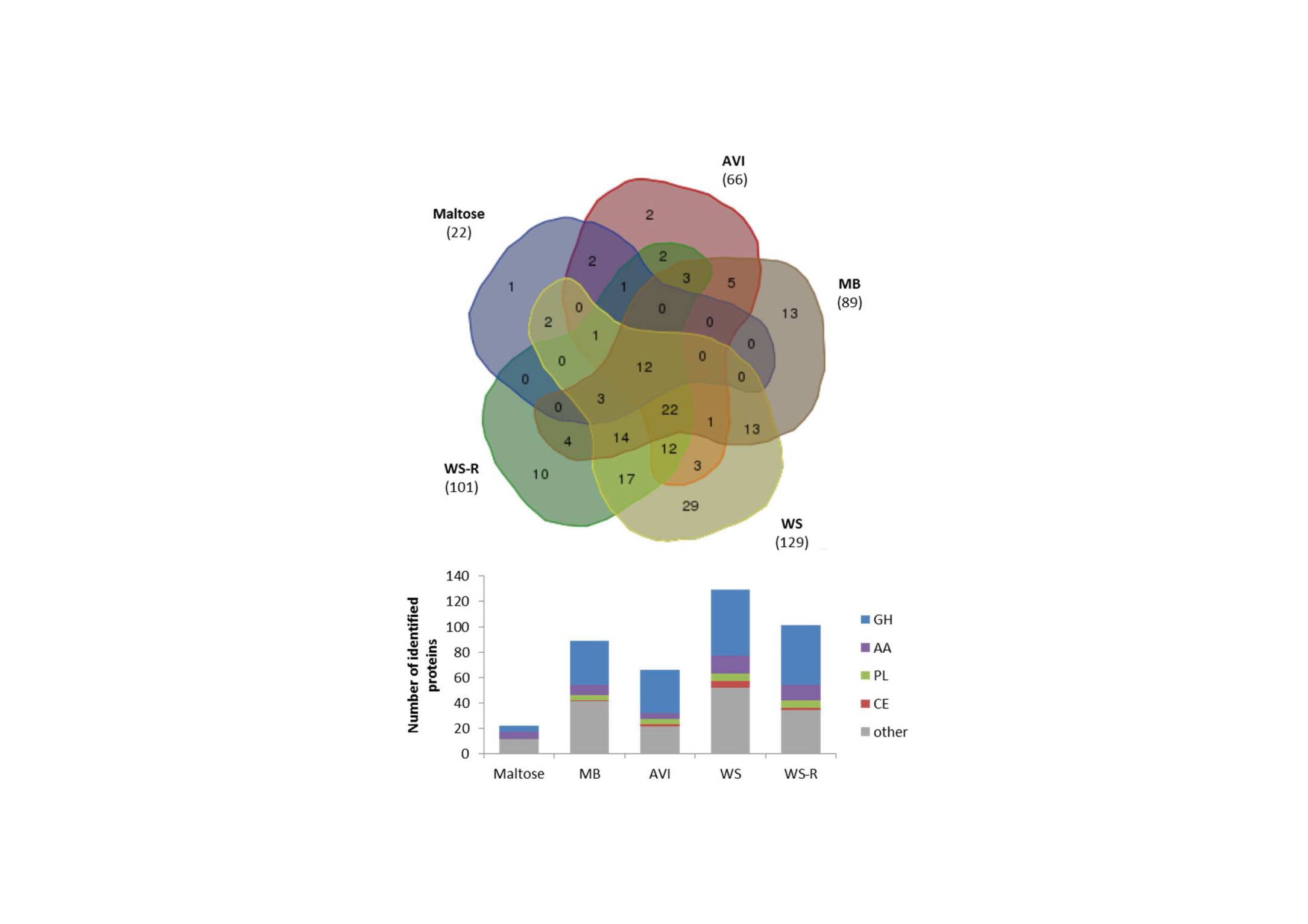
**

**Figure S6: Distribution of proteins identified by LC-MS/MS in *L. arvalis* secretomes from different growth conditions (maltose, maize bran [MB], Avicel [AVI], wheat straw [WS] and wheat-straw residue [WS-R]).** Top: Venn diagram showing the distribution of secreted proteins among the *L. arvalis* secretomes. Venn diagram was prepared using http://bioinformatics.psb.ugent.be. Bottom: Distribution profile of CAZymes in the secretomes. Bar size indicates the number of identified secreted proteins by class (GH, AA, PL, CE and other).

**Supplementary Tables:**

**Table S1: Sugar composition in % (w/w) of polysaccharides and biomass used for growth of *L. arvalis.***

|  | arabinose | xylose | mannose | galactose | glucose | total |
| --- | --- | --- | --- | --- | --- | --- |
| MB | 16 | 29 | <1 | 6 | 22 | 73 |
| AVI | 0 | 0 | 0 | 0 | 100 | 100 |
| WS | 3 | 21 | 1 | 1 | 35 | 61 |
| WS-R | 1 | 8 | <1 | 0 | 23 | 32 |

**Table S2: Main carbohydrate-cleaving activities of the *T. reesei* CL847 enzymatic cocktail and the secretomes of *L. arvalis* induced with Avicel (AVI), maize bran (MB), wheat straw (WS) and wheat straw residues (WS-R).** Enzyme activities are expressed in U.mg^-1^. Substrates abbreviations are listed in Fig. 4 legend. The substrates used were n.d., no activity detected.

|  | *T. reesei*  CL847 |  | *L. arvalis* CBS131.82 | | | |
| --- | --- | --- | --- | --- | --- | --- |
| Substrate |  |  | MB | AVI | WS | WS-R |
| pGlc | 0.22 ± 0.00 |  | 0.04 ± 0.00 | 0.08 ± 0.00 | 0.26 ± 0.00 | 0.15 ± 0.00 |
| pLac | 0.04 ± 0.01 |  | n.d. | 0.05 ± 0.00 | 0.01 ± 0.00 | 0.02 ± 0.01 |
| pCel | 0.06 ± 0.00 |  | n.d. | 0.06 ± 0.00 | 0.04 ± 0.00 | 0.02 ± 0.00 |
| pCel3 | 10.46 ± 0.53 |  | 0.86 ± 0.03 | 13.80 ± 0.30 | 10.10 ± 0.32 | 8.96 ± 0.33 |
| DCPIP | n.d. |  | 0.06 ± 0.11 | 0.84 ± 0.07 | 1.38 ± 0.25 | 1.44 ± 0.04 |
| CMC | 0.33 ± 0.02 |  | n.d. | 2.46 ± 0.08 | 0.93 ± 0.03 | 1.94 ± 0.12 |
| Avicel | 0.010 ± 0.005 |  | n.d. | 0.075 ± 0.004 | 0.010 ± 0.003 | n.d. |
| pXyl | 0.01 ± 0.00 |  | n.d. | 0.01 ± 0.00 | 0.01 ± 0.00 | n.d. |
| pAra | 0.03 ± 0.00 |  | 0.48 ± 0.01 | 0.01 ± 0.00 | 0.09 ± 0.00 | 0.03 ± 0.01 |
| pGal | n.d. |  | n.d. | n.d. | n.d. | n.d. |
| pMan | n.d. |  | n.d. | n.d. | n.d. | n.d. |
| Pect | 0.12 ± 0.00 |  | 6.73 ± 0.03 | 6.33 ± 0.16 | 0.77 ± 0.06 | 3.06 ± 0.09 |
| BirchX | 0.94 ± 0.04 |  | 0.10 ± 0.01 | 5.4 ± 0.51 | 4.39 ± 0.02 | 3.79 ± 0.10 |
| WheatX | 1.59 ± 0.03 |  | 0.35 ± 0.02 | 9.71 ± 0.80 | 5.93 ± 0.61 | 6.78 ± 0.02 |
| Man | 0.01 ± 0.00 |  | 0.06 ± 0.01 | 1.63 ± 0.08 | 1.10 ± 0.04 | 3.23 ± 0.23 |
| GMan | 0.02 ± 0.00 |  | 0.31 ± 0.06 | 3.99 ± 0.03 | 2.22 ± 0.10 | 6.88 ± 0.53 |
| Arab | 0.01 ± 0.00 |  | 0.14 ± 0.01 | 0.06 ± 0.02 | 0.23 ± 0.02 | 0.05 ± 0.01 |
| AraG | 0.01 ± 0.00 |  | 0.11 ± 0.05 | n.d. | n.d. | n.d. |

**Table S3: *In silico* identification of histidine methylations using LC-MS/MS.** Identifications were validated manually as described in material and methods.

|  | sequence | modification | e-value | MH+ obs | MH+ theo |
| --- | --- | --- | --- | --- | --- |
| contig11611 | HGGVTSYDIAGTK | H1:+14.0157 | 3.10E-04 | 1319.6605 | 1319.6595 |
| contig11611 | HGGVTSYDIAGTK | H1:+14.0157 | 8.40E-05 | 1319.6583 | 1319.6595 |
| contig12466 | HYIFTTLITPTTTSTAAVR | H1:+14.0157 | 7.30E-09 | 2108.1406 | 2108.1392 |
| contig12466 | HYIFTTLITPTTTSTAAVR | H1:+14.0157 | 3.40E-04 | 2108.1404 | 2108.1392 |

**Table S4: List of primers used to evaluate the expression levels of corresponding transcripts.**

| gene | transcript ID | primer ID | sequence (5’ -> 3’) |
| --- | --- | --- | --- |
| *actin-1* | contig09769 | ACT1F | aatgagctctcacggcagtt |
|  |  | ACT1R | gttcctgccactcttccttg |
| *LPMO* | contig08799 | PMO1F | gccagacgtggttcaagatt |
|  |  | PMO1R | gacgcggagaaggtattgac |
|  | contig12485 | PMO2F | ggtctggttcaaggtcaagg |
|  |  | PMO2R | tgcaatatgctccactcgaa |
|  | contig12466 | PMO3F | gcaaacttcaaccccttcaa |
|  |  | PMO3R | cgcatgagatgtaccactgc |
|  | contig12508 | PMO4F | attcctcctggccagtacct |
|  |  | PMO4R | gccagggaagctgactgtag |
|  | contig01004 | PMO5F | tgagctcatcgccattcata |
|  |  | PMO5R | aacaccagggtcagatgctt |
|  | contig11611 | PMO6F | gtgtacatggccaactgtgg |
|  |  | PMO6R | gggatcgtcgtcgtgtactt |
|  | contig11776 | PMO7F | ccacgagattcttggcctac |
|  |  | PMO7R | ggcagtatcgtctgggttgt |
| *CBH* | contig04291 | GH7-1F2 | ccctggtatcaaccgtggc |
|  |  | GH7-1R2 | gccggcagtcgacga |
|  | contig07647 | GH7-2F | gccaactataatgctgccgcttat |
|  |  | GH7-2R | tgaacttccggctcgtatcaat |
|  | contig08217 | GH7-3F2 | tggttctctcgctctcggtct |
|  |  | GH7-3R2 | gggagactgcgcctcg |
|  | contig08191 | GH7-4F | gcgccaaccgttacggt |
|  |  | GH7-4R | ccagacgcggtaccatcctt |
|  | contig08872 | GH7-5F2 | ccaagtttggcgaccagaactat |
|  |  | GH7-5R2 | ggttcacacctgggagtgaca |
| *CDH* | contig07486 | CDH1F | ATGCATCCCAATACCTCAAGAGT |
|  |  | CDH1R | GACGTTGAGGCCATCAGGG |
|  | contig08138 | CDH2F | GACACAGTACCTGCGCGA |
|  |  | CDH2R | TGAGAGGTGAACGCGAATCC |
